# Kinematics and kinetics of the shoe during unexpected ladder slip events

**DOI:** 10.1016/j.jbiomech.2025.112798

**Published:** 2025-06-03

**Authors:** Violet M. Williams, Sarah C. Griffin, Mark S. Redfern, Kurt E. Beschorner

**Affiliations:** University of Pittsburgh, 303 Schenley Place, Pittsburgh, PA 15213, USA

**Keywords:** Shoe dynamics, Ladder climbing, Slips, trips, and falls, Ground reaction forces

## Abstract

Foot slipping is a common initiating event in ladder falls, often leading to severe injuries. Methods for measuring the slipperiness of rungs are impeded by a lack of data on slip events during climbing. This study investigated the kinematics and kinetics of ladder slip events to guide coefficient of friction test methods for ladder rungs. Eighty-eight participants (45F, 43 M; 43.4 ± 12.3 yrs; 80.1 ± 16.9 kg; 170.9 ± 91.6 cm) climbed a series of ladder configurations with differing rung designs and ladder angles in a randomized order. A liquid contaminant (90 % glycerol +10 % water by volume) was applied during each participant’s final trial and the responses were categorized into slip and non-slip events with a cluster analysis and reviewer classification. A total of 11 slipping events (9 forward and 2 backward slips) were identified from 102 ladder climbs based upon their peak slipping speeds and slip distances. Slipping speed, shoe-rung angle, transverse foot angle, normal force, shear force, anterior-posterior (AP) foot position, and medio-lateral (ML) foot position were quantified at the start of slip and at the peak slipping speed for forward slips with mean values and 95 % confidence intervals reported. Slips had speeds of 0.34 m/s at their peak, with normal forces around 460 N. We argue the need for testing conditions informed by the under-shoe conditions observed in this study: forward slipping (8° dorsiflexion, 0.34 m/s, 400 N).

## 1. Introduction

Falls are a common cause of injury that accounted for 211,000 US workplace injuries between 2021–2022 (U.S. Bureau of Labor Statistics, 2024b). Falls from height are some of the most severe falls, comprising 83 % of fall-related fatalities (U.S. Bureau of Labor Statistics, 2024a). Ladders accidents account for 30 % of falls from height (U.S. Bureau of Labor Statistics, 2024b). While the factors contributing to ladder falls are diverse, slip events account for 14 % (Cohen and Lin, 1991). As the study of ladder slipping events is an emerging area of research, risk factors and assessment tools relevant to ladder slip risk are still being identified and developed.

Recent work has identified factors that impact slip risk during perturbed and unperturbed ladder climbing, such as biomechanical factors surrounding climbing style (Pliner and Beschorner, 2017; Pliner et al., 2014; Pliner et al., 2019), foot orientation (Griffin et al., 2023; Griffin et al., 2025; Martin et al., 2020; Pliner et al., 2014; Pliner et al., 2019; Williams et al., 2024), and body orientation (Griffin et al., 2023; Griffin et al., 2025; Martin et al., 2020; Pliner et al., 2014) with a goal of determining safe climbing techniques. Additional research has focused on the impact of ladder design factors, such as ladder angle (Martin et al., 2020), extension ladder configuration (Williams et al., 2024), and roof-to-ladder transition attachments (Griffin et al., 2023), on slip risk or slip severity. The design of higher traction footwear and ladder rungs is a potential avenue towards reducing slip risk in ladder climbing. Despite current requirements that metal ladder rungs are designed to minimize slipping (e.g. “corrugated, knurled, dimpled, coated with skid-resistant material, or otherwise treated to minimize slipping”) (Occupational Safety and Health Administration, 2014), friction testing methods for ladder rungs are not developed.

Mechanical slip testing is used to measure the available coefficient of friction (ACOF) between two surfaces. This testing can determine how slip risk varies across shoes (Blanchette and Powers, 2015a; Grönqvist, 1995; Gupta et al., 2022a; Gupta et al., 2022b; Jones et al., 2018; Walter et al., 2021; Wilson, 1996) and flooring design (Beschorner et al., 2017; Beschorner and Randolph, 2023; Chanda et al., 2018; Chanda et al., 2019; Gupta et al., 2022c). Testing standards help minimize the effects of testing conditions known to affect ACOF outcomes such as sliding speed (Beschorner et al., 2007; Blanchette and Powers, 2015b; Redfern and Bidanda, 1994), contact time (Grönqvist et al., 2003), vertical force (Beschorner et al., 2007; Blanchette and Powers, 2015b), and shoe-floor angle (Beschorner et al., 2007; Blanchette and Powers, 2015b). Ideally, standard test conditions should mimic real slip events to achieve biofidelic tests that are relevant to human slips (Albert et al., 2017; Chang et al., 2001; Iraqi et al., 2018). However, there is a paucity of research characterizing slip events during ladder climbing.

Currently, there are no standard slip testing procedures established for ladder rungs unlike level walking, where extensive biomechanical studies have informed biofidelic friction testing methods (Albert et al., 2017; Cham and Redfern, 2002; Chambers et al., 2003; Iraqi and Beschorner, 2017; Iraqi et al., 2018; McGorry et al., 2010; Moyer et al., 2006; Strandberg and Lanshammar, 1981). This level-walking literature has included metrics such as slipping speed (Albert et al., 2017; Cham and Redfern, 2002; Chambers et al., 2003; Iraqi and Beschorner, 2017; Iraqi et al., 2018; McGorry et al., 2010; Moyer et al., 2006; Strandberg and Lanshammar, 1981), shoe-floor angle (Albert et al., 2017; Cham and Redfern, 2002; Chambers et al., 2003; Iraqi et al., 2018; McGorry et al., 2010; Moyer et al., 2006; Strandberg and Lanshammar, 1981), normal force (Iraqi and Beschorner, 2017; Iraqi et al., 2018; Strandberg and Lanshammar, 1981), and contact time (Cham and Redfern, 2002; Iraqi et al., 2018; Strandberg and Lanshammar, 1981) throughout the slip event, including the start of slip and peak slipping speed. Additional work has also compared the kinematics/kinetics of expected and unexpected slipping events (Iraqi et al., 2018), to determine whether current standards accurately reflect the unexpected nature of slips in the workplace. While this established literature has improved our understanding of slips in level walking, ladder slip events represent an emerging area in literature that is still being characterized. As mentioned previously, recent research has begun characterizing the biomechanical factors relevant to slip and misstep risk (Griffin et al., 2025; Martin et al., 2020; Pliner et al., 2014). However, these studies have not characterized shoe dynamics at key moments in the slipping phase and, therefore, are insufficient to guide friction testing. Thus, quantifying these factors for ladder slipping events is important to informing rung-shoe friction test methods.

The aims of this study were to 1) quantify biomechanical variables of unexpected slips during ladder climbing and 2) determine biofidelic testing conditions for mechanical slip testing of ladder rungs. To accomplish these goals, we will describe the central tendencies and confidence intervals of these biomechanical parameters and develop suggested biofidelic testing conditions based upon them.

## 2. Methodology

### 2.1. Participants

Eighty-eight participants (45 female, 43 male; 43.4 ± 12.3 years old; 80.1 ± 16.9 kg; 170.9 ± 9.2 cm) were enrolled in the study based upon an *a priori* power analysis completed for the broader study (logistic regression that assumed an odds ratio of 3 for 1 standard deviation shift in the regressor; α = 0.008; β = 0.2; average slip rate of 0.5). Participants were eligible if they were 18–65 years, climbed ladders at least 10 times in the previous year, weighed under 136 kg (due to harness system restrictions), had a height of less than 196 cm (due to ceiling restrictions), had a BMI under 35, and were generally healthy. Ethical approval was obtained from the University of Pittsburgh Institutional Review Board (Study 19100204) and all participants provided informed consent. This research was performed in accordance with the Declaration of Helsinki of 1975.

### 2.2. Instrumentation

**Fig. 1.**
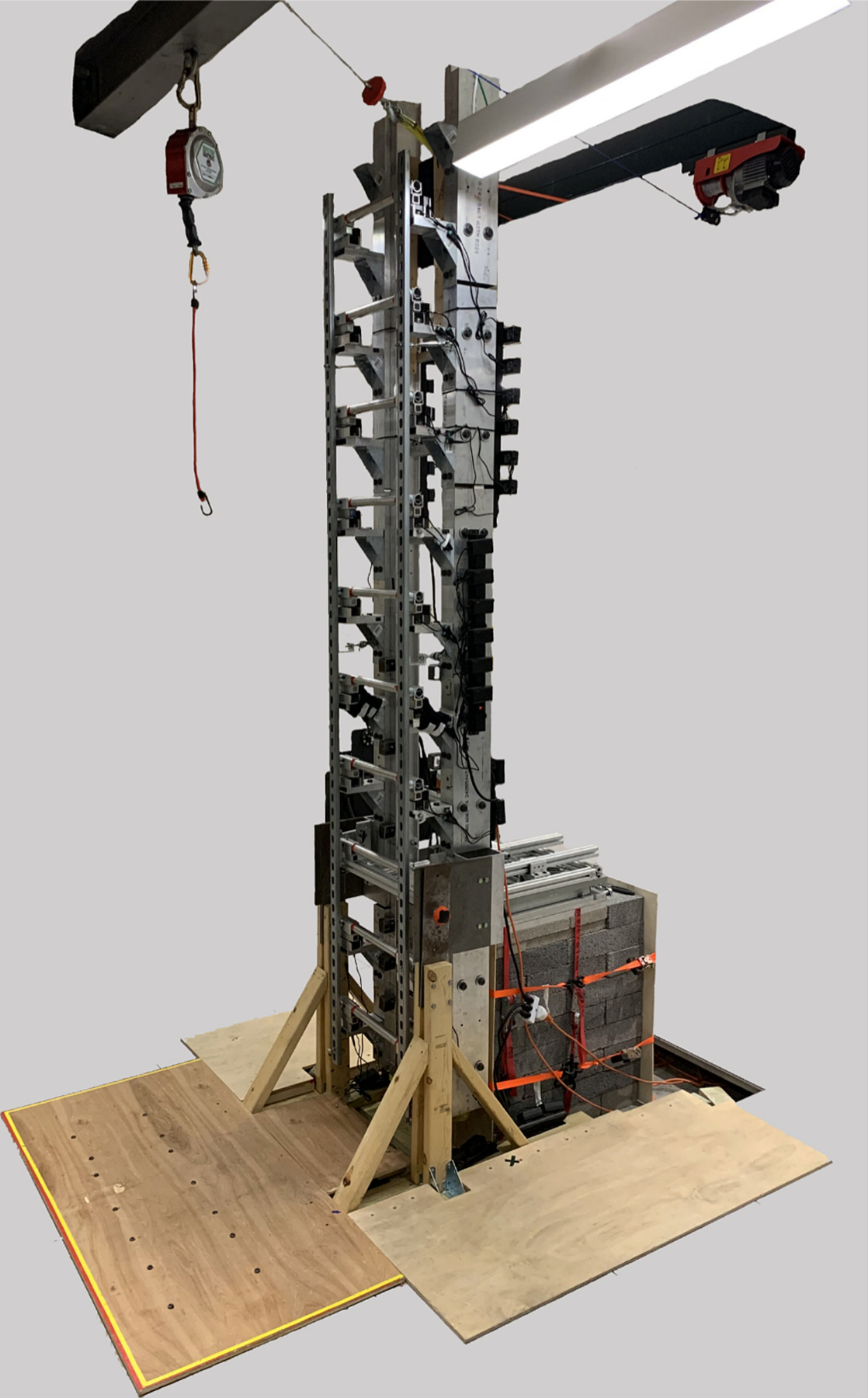
The ladder apparatus with interchangeable rungs and the ability to rotate to three discrete angles. The third rung was detached from the rest of the ladder and attached to a force plate located behind the ladder.

This study used a custom instrumented ladder apparatus which could rotate to three discrete angles and had interchangeable rungs (Fig. 1). The third rung (chosen to ensure steady-state climbing) of the ladder was rigidly attached to a force plate (AMTI Inc., Watertown, MA, USA; 1080 Hz) to collect kinetic data at the shoe-rung interface, while 12 motion tracking cameras (Vicon Motion System Ltd., Centennial, CO, USA: 120 Hz), positioned around the ladder apparatus, collected time-synced kinematic data. A set of seventy-nine reflective markers (Moyer, 2006) were secured to anatomical landmarks. This anatomical marker set, required to capture full-body kinematics for the broader study, included five foot markers relevant to the foot kinematics investigated here: a medial heel marker (inferior to the medial malleolus), a lateral heel marker (inferior to the lateral malleolus), an inferior heel marker (inferior on the posterior of the calcaneus), a medial toe marker (distal end of the hallux), and a lateral toe marker (distal end of the 4th phalange). Participants were outfitted with tight fitting athletic wear, helmet, shin guards, knee pads, elbow pads, gloves, shoes (Vans Authentic Wide Shoe, Cost Mesa, CA), and a safety harness attached to a fall arrest system (Self Retracting Lanyard, Ropes Park Equipment, Fairfield, CT, USA). Participant height and weight were recorded at the beginning of testing with and without all the worn equipment.

### 2.3. Procedure

**Fig. 2.**
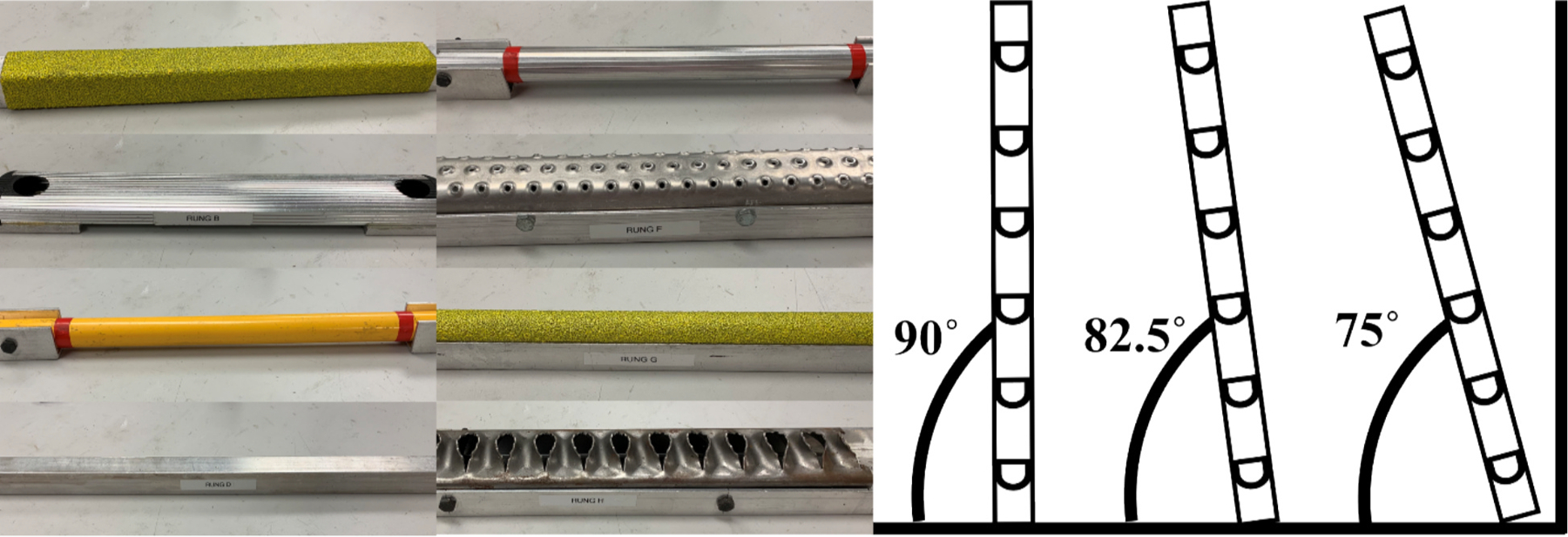
Participants encounter eight different rungs of differing size, shape, and slip resistance classifications. The ladder was oriented at three different angles relative to horizontal.

Participants climbed a series of ladder configurations consisting of eight unique rung designs and three angles (Fig. 2). The order of configurations was randomized for each participant to ensure that a range of ladder rungs and ladder angles were encountered in the slippery condition. Three climbing trials were completed in each configuration. Participants were instructed to climb up the ladder urgently, stopping at the 5th rung, and then descend the ladder. Climbing speed was not constrained, to ensure the generalizability of this work across physical ability levels. During the last configuration, participants climbed the ladder a fourth time, for their final trial, in which a liquid contaminant (90 % glycerol +10 % water by volume) was added to the third rung. Participants completed a word puzzle task to distract them from contaminant application and ensure the slip was unexpected. Some participants (n = 46) completed this task on the fifth rung of the ladder and the contaminant was applied prior to their ladder descent. The remaining participants (n = 42) completed this task on the ground while facing away from the ladder allowing for contaminant application prior to their ladder ascent. To increase the slipping rate, 59 participants climbed a ladder with plexiglass plates installed in front of the rung to limit toe clearance (Pliner, 2020; Pliner et al., 2014). The lights were dimmed to prevent subjects from noticing the contaminant.

### 2.4. Data analysis

Perturbation trials were included in the analysis if the participant was unaware of the contaminant prior to their climb. For participants who had the contaminant applied prior to their ladder ascent, the descending perturbation trial was also included if the participant did not notice the contaminant after the ascending climb and did not have a slip during the ascent (Albert et al., 2017; Iraqi and Beschorner, 2017; Iraqi et al., 2018).

**Fig. 3.**
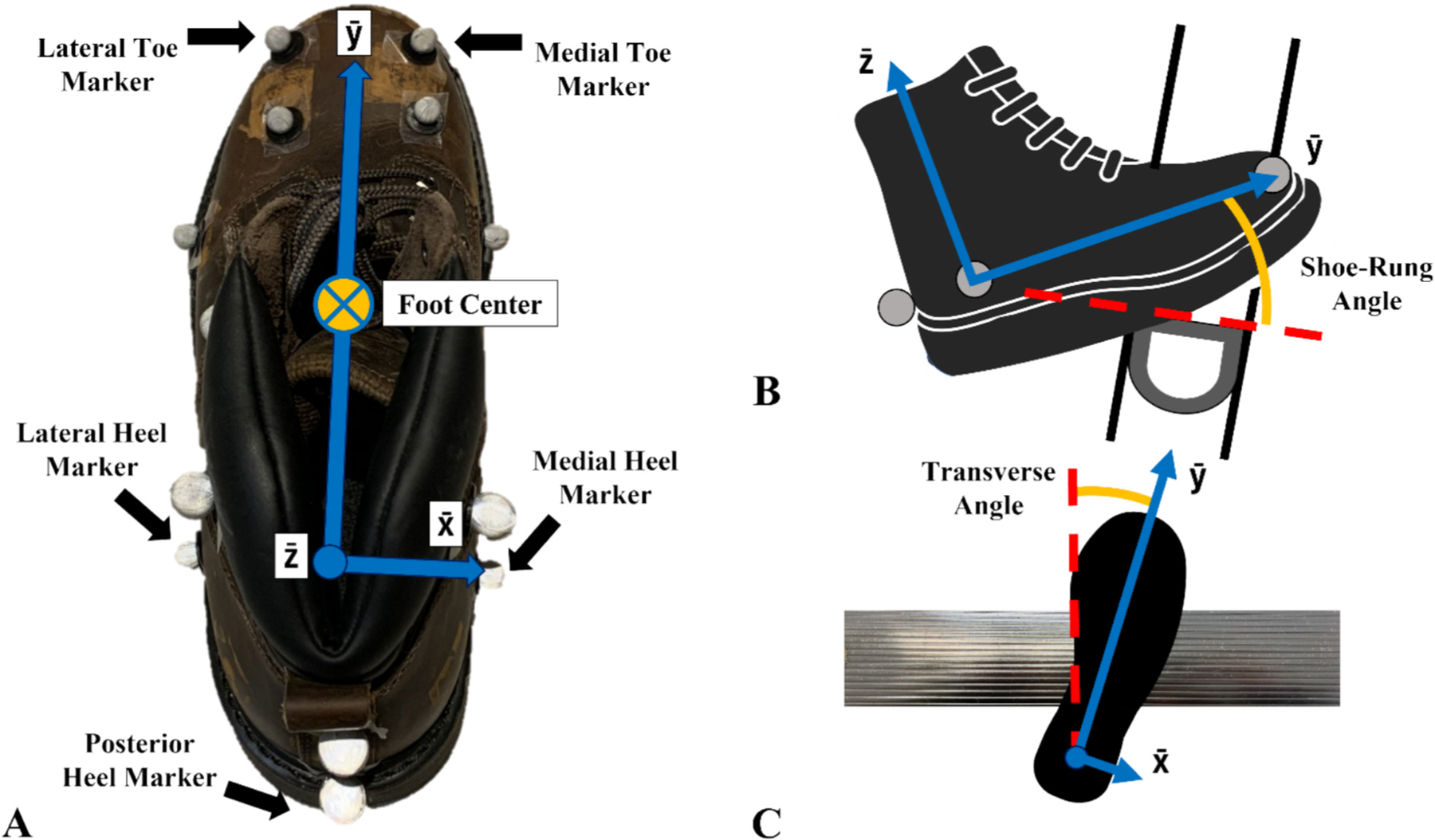
**A)** The foot local coordinate system with the relevant foot markers and the foot center which is calculated from the medial/lateral toe and heel markers. **B)** Shoe-rung angle is the angle between the long axis of the foot and the rung surface where 0° occurs when the foot is perpendicular to the ladder plane. **C)** Transverse foot angle is defined as the angle between the long axis of the foot and the rung where 0° occurs when the foot is perpendicular to the rung.

**Fig. 4.**
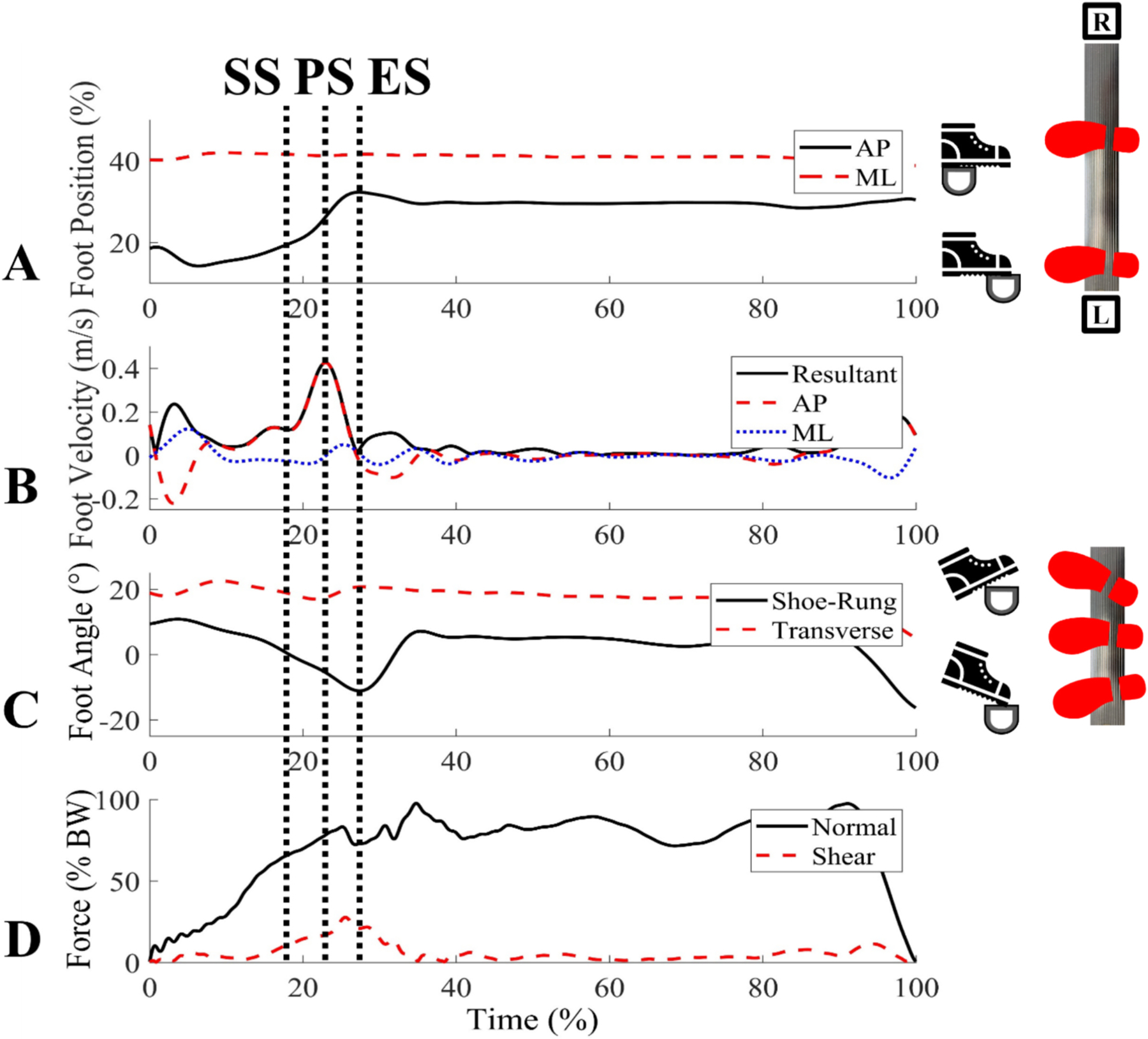
Kinematic and kinetic measures for one representative trial. In each plot, the times corresponding with the start of slip (SS), peak slipping speed (PS), and end of slip (ES) are labeled. These metrics include **A)** Foot position where AP position is a solid black line and ML position is a dashed red line. **B)** Foot velocity where resultant velocity is a solid black line, AP velocity is dashed red line, and ML velocity is a dotted blue line. **C)** Foot angle where shoe-rung angle is a solid black line and transverse foot angle is a dashed red line. **D)** Force where normal force is a solid black line and shear is a dashed red line.

A local coordinate system was defined for the foot using four anatomical markers. An x vector pointed to the right with an origin defined as the midway point between the medial and lateral heel markers. A z vector, perpendicular to a plane formed by the x vector and an anterior vector pointing from the midpoint of the heel markers to the midpoint of the front foot markers, pointed superiorly. A y vector, orthogonal to the x vector and z vector, pointed anteriorly. (Fig. 3a). The foot position while standing in a static reference orientation was used to define a neutral (0°) position for the shoe plane in the global system. The foot center was calculated as the average position of the medial and lateral heel markers and the two front foot markers (Fig. 3a). This local coordinate system and foot center were used to define slipping speed, normal force, shear force, shoe-rung angle, transverse foot angle, foot position, and slip distance. These variables (except slip distance) were quantified at the start of the slip and the time of peak slipping speed (Fig. 4). Slipping speed was the resultant velocity of the foot center along the shoe-rung plane (defined by the shoe angle). Peak slipping speed was the maximum resultant speed of the foot center during contact. Local minima around the peak slipping speed defined the start and end of slip. Slipping speed (resultant velocity), anterior posterior (AP) velocity, and medial–lateral (ML) velocity were reported. Normal force was the force component perpendicular to the sole (z-axis, Fig. 3) in the local foot coordinate system. Shear force was the resultant of the force components within the plane of the foot (x and y-axis, Fig. 3). Shoe-rung angle was calculated as the angle, between the long axis of the foot and the rung surface in the sagittal plane where 0°corresponds to the foot being perpendicular to the ladder plane (Fig. 3b). Transverse foot angle was calculated as the angle between the foot and the rung in the transverse plane, with 0°corresponding to the foot being perpendicular to the ladder rung, and a positive value indicating that the toe is rotated laterally from this orientation (Fig. 3a). AP foot position was quantified as a percentage of foot length based on the rung position relative to the foot, where 0 % corresponds to contact at the medial toe marker and 100 % corresponds to contact at the posterior heel marker. ML foot position was quantified as the point along the rung where shoe contact was made, where 0 % corresponds to the foot center being at the leftmost point of the rung and 100 % corresponds to the foot center being at the rightmost point of the rung. Slip distance was the displacement of the foot center between the start of slip and the end of slip, defined as the local minima in resultant velocity just after peak slipping speed.

Slips were classified using a k-means cluster analysis that grouped the trials based on the peak slipping speed and slip distance (Beschorner et al., 2016; Chambers and Cham, 2007; Jones et al., 2018; Moyer et al., 2006). Both parameters were transformed into z-score distributions to prevent variable scales from influencing the clustering. This analysis minimizes the sum of distances between data points and cluster centroids with the algorithm iterating through groupings until this minimization is complete. The Davies-Bouldin Index and the Silhouette score were used to determine the optimal number of groups for this classification. The Davies-Bouldin index determines how well clusters are separated in a model and the Silhouette determines how well-defined clusters are based upon inter-cluster distances and intra-cluster distances. Two reviewers, with an inter-rater reliability of 93 %, observed each slip trial classified by the cluster analysis to confirm each classification or remove it from the slip group if it was found to be incorrectly classified. The cluster analysis and reviewer observations provided both objective and subjective classification methods that increased confidence in the final slip classifications. Mean values and 95 % confidence intervals were calculated for all metrics (slipping speed, shoe-rung angle, transverse foot angle, normal force, shear force, AP foot position, and ML foot position) at the time of slip start and peak slipping speed.

## 3. Results

### 3.1. Cluster analysis

**Fig. 5.**
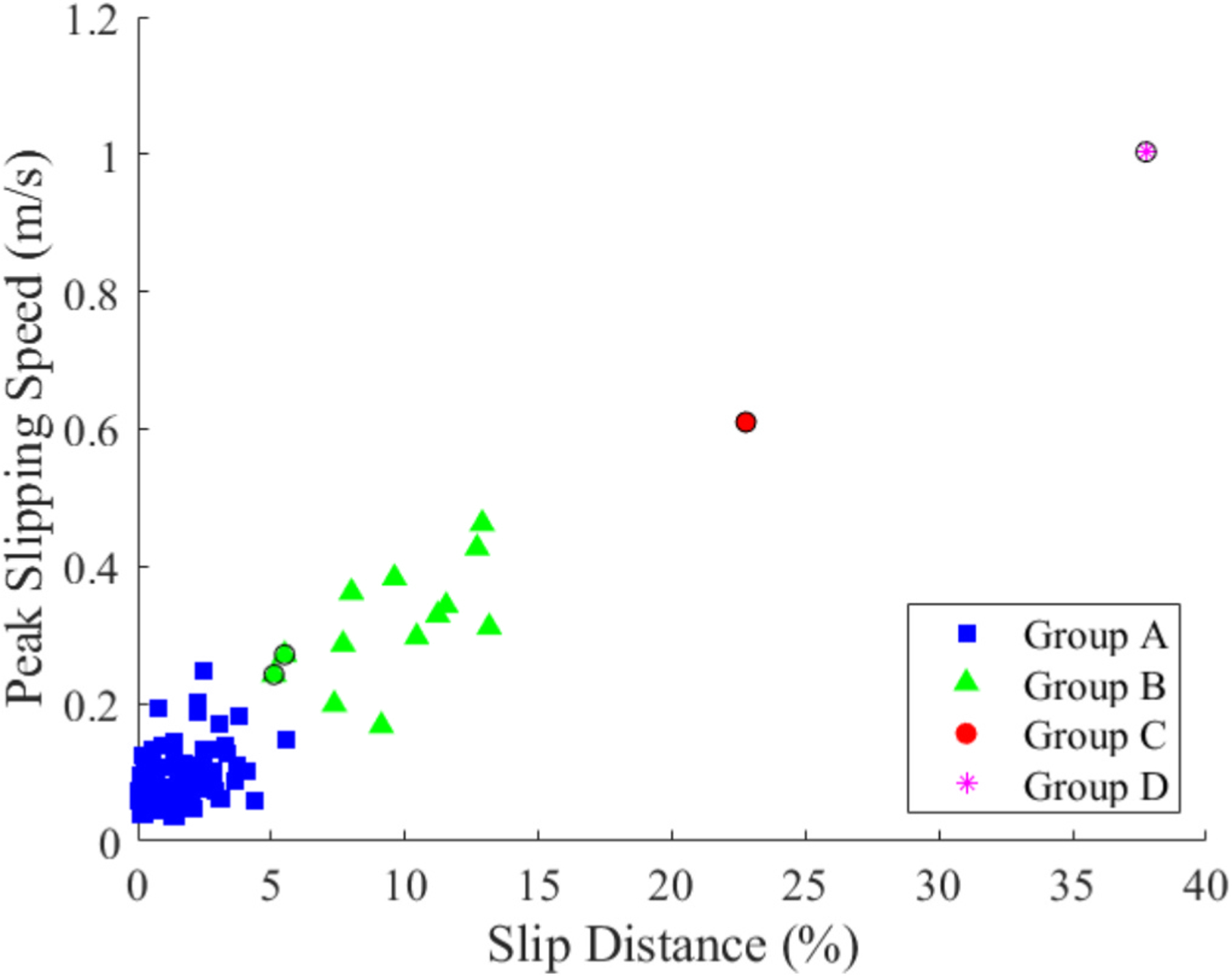
Cluster analysis groupings of slip trials shown with peak slipping speed vs slip distance. The four groups (A, B, C, D) are based on the results of the cluster analysis. Four trials were categorized as non-slip events (noted with a thick black outline) after further review. The points in Groups C and D were later identified as the motion when the foot was stepping off of the rung and were not counted as slip events.

**Fig. 6.**
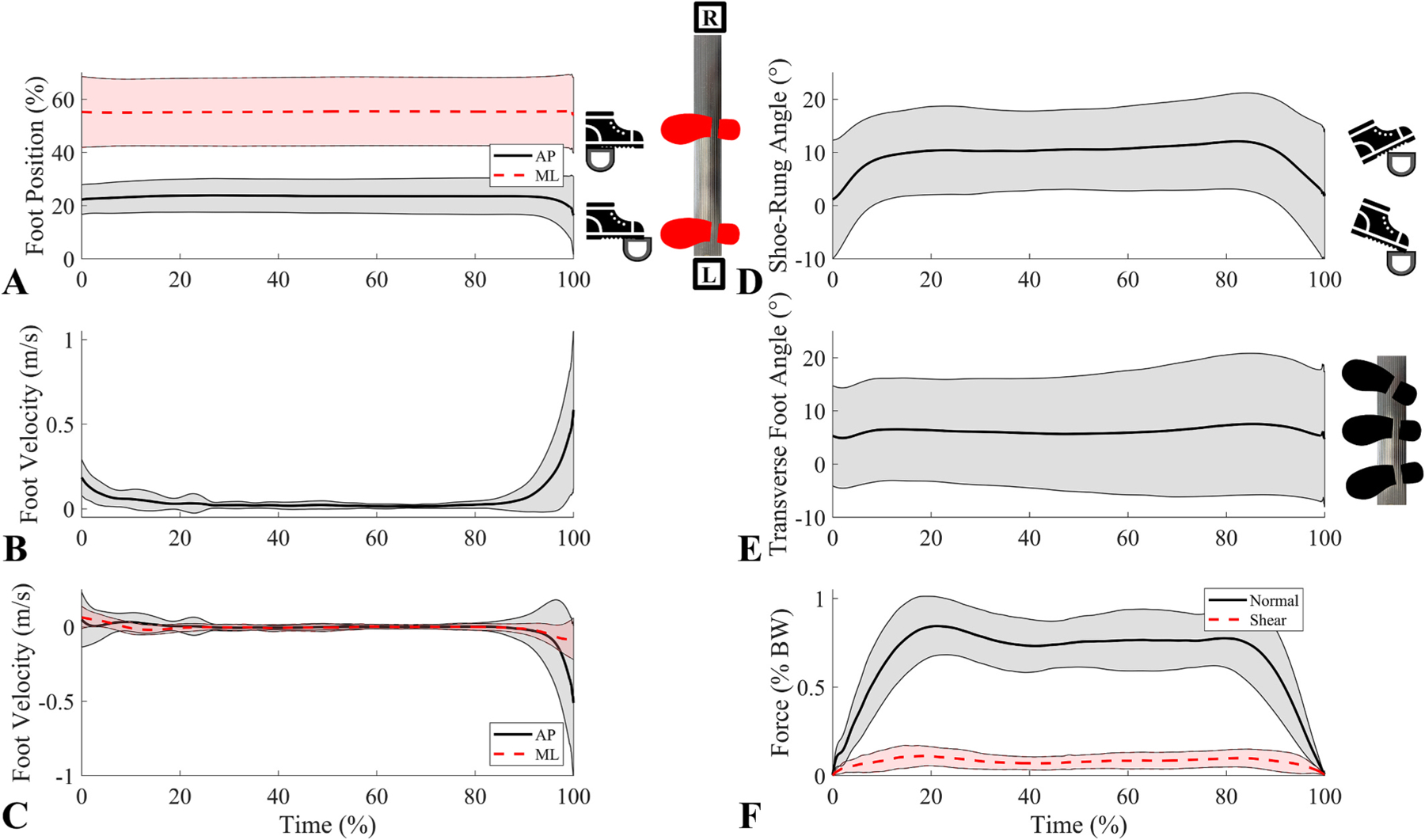
Average kinematic and kinetic measures for the non-slip trials along with their standard deviations (shaded regions represent regions within 1 standard deviation of the mean). The x-axis is % time on the rung (0 % at contact and 100 % at lift-off) These metrics include **A)** Foot position where AP position is a solid black line and ML position is a dashed red line. **B)** Resultant foot velocity. **C)** Foot velocity where AP velocity is a solid black line and ML velocity is a dashed red line. **D)** Shoe-rung angle. **E)** Transverse foot angle. **F)** Force where normal force is a solid black line and shear is a dashed red line.

The Davies-Bouldin Index (0.31) and the Silhouette score (0.90) returned an optimal grouping of four clusters based on slip distance and peak slipping speed (Fig. 5). The k-means cluster analysis minimized the total sum of distances to 21.2 over three iterations. Group A consisted of low peak slipping speed values (0.09 ± 0.04 m/s) and low slip distance values (1.54 ± 1.21 % of foot length). Group B consisted of higher peak slipping speed values (0.31 ± 0.08 m/s) and higher displacements (9.56 ± 2.72 % of foot length). Groups C and D each contained one individual point, both of which had high peak slipping speeds (0.61 & 1.00 m/s) and slip displacement (22.77 & 37.74 % of foot length). As Groups C and D each only had a single point and were characterized by more severe slipping (higher speed and larger distance), these two clusters were combined with Group B. These grouped points (B, C, & D) were classified as slips, resulting in a total of 15 slip events (Fig. 5). Group A was classified as non-slips, resulting in a total of 87 non-slip trials (Fig. 6). Of the 15 slips identified by the cluster analysis, 4 were removed following the reviewer analysis, resulting in 11 slips.

### 3.2. Kinematics/kinetics

Of the eleven slip trials, nine were forward slips and two were backward slips. Due to the low number of backward slips, kinematic and kinetic averages and confidence intervals were only calculated from the nine forward slips. Forward slips primarily occurred in early stance with a mean slip time that was 11.5 ± 15.2 % into stance. Forward slips had a mean slipping speed of 0.05 m/s at slip start and 0.34 m/s at the peak slipping speed (Table 1). The mean AP component was 0.33 ± 0.08 m/s and the mean ML component was −0.02 ± 0.08 m/s, indicating that slips were primarily in the AP direction. The average total slip displacement was 28.1 ± 4.6 mm. Forward slips had mean shoe-rung angles of 7.6 ± 7.6° at the start of slip and 7.0 ± 4.5° at peak slipping speed, resulting in slightly dorsiflexed foot orientations for both time points. Normal forces were around half of bodyweight for both time points, with means of 38.3 ± 34.8 % BW (288.9 ± 240.8 N) at the start of slip and 58.6 ± 24.6 % BW (460.1 ± 224.5 N) at peak slipping speed. The remaining kinematic and kinetic metric means and confidence intervals can be found in Table 1. The two backward slips had lower peak sliding speeds (0.20 m/s & 0.29 m/s), lower slip displacements (17.9 mm & 17.5 mm), and more dorsiflexed shoe-rung angles (21.4° & 20.6°) compared to the forward slip averages.

## 4. Discussion

This work aimed to address the lack of standard testing parameters for ladder rung ACOF testing by characterizing the biomechanical variables of ladder slip events necessary for defining biofidelic conditions. Our results provided new information on the kinematics and kinetics of ladder slip events needed to define these conditions. Ladder slip events had lower slipping speeds (0.05 m/s) at the start of slipping that rise to a mean of 0.34 m/s at peak slipping speed. Slips occurred primarily in the AP direction with a neutral shoe-rung angle that is only slightly dorsiflexed (7.0°) on the rung and a slight transverse foot angle (9.5°) that rotates the toes laterally. The rung contacted the shoe around the ball of the foot (23.0 %) before sliding, on average, 28 mm forward, with foot positions occurring near the center of the ladder rung mediolaterally (55.5 %). On average, normal forces were half of bodyweight (58.6 % BW) and were about five times greater than shear forces (13.2 % BW).

This study was consistent with other studies reporting kinematics during ladder slip events (Pliner et al., 2014). The average AP foot position (23 %) was similar to previous published results (20 %) investigating slip outcomes in straight ladder climbing (Pliner et al., 2014). The average shoe-rung angles during ascent (13° dorsiflexed) and descent (7° dorsiflexed) were similar to previous results investigating slip outcomes during ascent (12° dorsiflexed) and descent (−2° plantarflexed) in straight ladder climbing (Pliner et al., 2014).

Previous steady-state ladder climbing studies can further contextualize these results (Bloswick and Chaffin, 1990; Griffin et al., 2023; Griffin et al., 2025; Martin et al., 2020; Williams et al., 2024). While the 38.3 % BW mean normal force observed at slip onset was lower than previous findings of 50–60 % BW mean vertical (approximately the normal direction) foot forces averaged throughout stance, the 58.6 % BW normal force at peak slipping speed was within this range (Bloswick and Chaffin, 1990). Slipping may start before the foot is fully loaded. The shear forces observed at slip onset in this study (7.1 % BW) were comparable with the mean reported horizontal forces during steady-state climbing (10–20 % BW). Most slips occurred in early stance supporting literature’s focus on frictional requirements during early stance (Griffin et al., 2023; Griffin et al., 2025; Martin et al., 2020; Williams et al., 2024). However, the existence of one mid-stance slip (48.5 % stance time) indicates potential need to investigate frictional requirements during later stance (Williams et al., 2024). Additionally, our results showed that forward slips occurred with slightly dorsiflexed foot angles (7.6°), in agreement with one previous study that found higher frictional requirements with dorsiflexed shoe-rung angles during ascent (Martin et al., 2020). However, three other studies found increased plantarflexion associated with higher frictional requirements during descent (Griffin et al., 2023; Griffin et al., 2025; Williams et al., 2024) though two (Griffin et al., 2023; Williams et al., 2024) involved ladder transitions (roof-to-ladder and extension ladder) rather than typical steady-state climbing. Thus, this study is largely consistent with prior research in terms of the under-shoe conditions and the potential for slipping during early and late stance.

Slip kinematics and kinetics are important for informing future mechanical slip testing of ladder rungs. Current ACOF testing parameters for slips during level walking operate with sliding speeds of 0.3 m/s and 0.5 m/s, normal forces between 250 and 500 N, and shoe-floor angles of 0° and 17° dorsiflexed (American Society for Testing and Materials, 2024; International Organization for Standardization, 2019; National Floor Safety Institute, 2021). In ladder climbing, the mean peak slipping speed (0.34 m/s) is similar to the parameters for walking. The average normal forces found in ladder slip kinematics were 289 N and 460 N during the start of slip and peak slipping, respectively, indicating that, like in walking, a normal force condition of 400 N would be sufficient (American Society for Testing and Materials, 2024; International Organization for Standardization, 2019). This study suggests that an 8° dorsiflexed orientation for forward slips would be appropriate. Based on these results, we recommend friction testing that approximates a sliding speed of 0.34 m/s, a normal force of 400 N, and a contact angle of 8° dorsiflexed. Such conditions would lead to rung friction measurements that are biomechanically relevant to ladder climbing.

### 4.1. Limitations

One limitation of this study is the low number of slip events. This primarily limits comparisons between slipping direction because there were only 2 backward and 9 forward slips. Another limitation is the use of a single liquid contaminant and shoe design, limiting the generalizability of these results to other shoes and contaminants (Chander et al., 2017; Jones et al., 2018). Finally, the laboratory setting may have caused participants to climb differently than they would in typical outdoor settings, which may have impacted their climbing kinematics/kinetics.

## 5. Conclusion

This study provides the kinematic and kinetic measures needed to better understand slip events during ladder climbing. These measures suggest speed (0.34 m/s), force (400 N), angle (8° dorsiflexed), and slipping direction (forward) that can be used to develop a series of biofidelic mechanical ladder rung slip testing procedures.

## Figures and Tables

**Table 1:** Slip trial kinematic & kinetic means and confidence intervals for the mean at the start of slip and the peak slipping speed.

	Slip Start		Peak Slipping Speed	
Metric	Mean	95 % Confidence Interval	Mean	95 % Confidence Interval
**Slipping Speed (m/s)**	0.05	0.02–0.07	0.34	0.28–0.41
**Shoe-Rung Angle (°)**	7.6	1.7–13.5	7.0	3.6–10.5
**Transverse Foot Angle (°)**	7.6	1.6–13.7	9.5	2.1–16.8
**Normal Force (% BW)**	38.3	11.6–65.0	58.6	39.7–77.4
**Shear Force (% BW)** *	7.1	0.01–0.10	13.2	6.7–19.8
**AP Foot Position (%)**	23.0	20.5–25.5	28.6	30.9–26.3
**ML Foot Position (%)**	55.2	47.2–63.2	55.5	47.6–63.4

* These values were log transformed to calculate the confidence interval.
